# Does the use of Surgicel affect postsurgical radiological follow-up of kidney tumors?

**DOI:** 10.1016/j.radcr.2024.03.089

**Published:** 2024-04-24

**Authors:** Anahita Ansari Djafari, Seyyed Ali Hojjati, Amir Hossein Eslami, Sina Samenezhad, Saba Faraji

**Affiliations:** a Urology Department, School of Medicine, Shahid Beheshti University of Medical Sciences, Tehran, Iran; b Laser Application in Medical Sciences Research Center, Shahid Beheshti University of Medical Sciences, Tehran, Iran; c Men's Health & Reproductive Health Research Center, Shahid Beheshti University of Medical Sciences, Tehran, Iran; d Assistant Professor of Psychiatry, Tehran University of Medical Sciences, Tehran, Iran

**Keywords:** Oxidized cellulose, Surgicel, Nephrectomy

## Abstract

Remaining Surgicel in the body can be mistaken for complications such as hematoma, abscess, or tumor recurrence in paraclinical examinations after surgery. We have presented the case of kidney cancer who underwent radical nephrectomy. In radiological follow-ups, hematoma was reported in the surgery site. The typical appearance of Surgicel on a postoperative CT scan is characterized by air trapped bubbles. Surgicel exhibits a short relaxation time on T2-weighted images. It is important to differentiate the remaining Surgicel from cases such as hematoma, abscess and tumor recurrence. T2MRI images will be most accurate in the correct diagnosis of Surgicel.

## Introduction

Oxidized regenerated cellulose, commonly known as Surgicel, is a type of absorbable hemostatic agent commonly used in surgical procedures to effectively manage and control bleeding [1]. When Surgicel becomes saturated with blood, it aids in the formation of a clot, and within 2 to 8 minutes, it can stop bleeding and create hemostasis [2]. In rare cases, complications such as infection and allergic reactions have been reported following the use of Surgicel [3]. Surgicel is typically absorbed and disappears from the body within 1 to 8 weeks, but in some cases, its absorption can be delayed. Remaining Surgicel in the body and its lack of absorption can be mistaken for complications such as hematoma, abscess, or tumor recurrence in paraclinical examinations after surgery, such as CT scan and MRI. As follow-ups with CT scan and MRI will be required in kidney cancer surgeries, accurately diagnosing the presence of Surgicel and distinguishing it from a hematoma, abscess, or tumor recurrence is crucial [4]. In this article, we present a case of renal tumor who underwent radical nephrectomy, and residual Surgicel was observed which mostly mimics the manifestations of hematoma.

## Case report

A 72-year-old woman presented with a mass measuring 52 × 58 × 76 mm with heterogeneous enhancement that contained areas of necrosis and neovascularization in the middle and lower pole of her right kidney. During the investigation for metastatic disease, no evidence of metastasis was identified. The patient underwent a right radical nephrectomy, and surgicel was placed in the surgical site. The pathological examination revealed clear cell renal cell carcinoma (RCC) with a tumor stage of pT2NxMx. The surgical margins were clear, and there was no lymphovascular invasion.

**Fig. 1:**
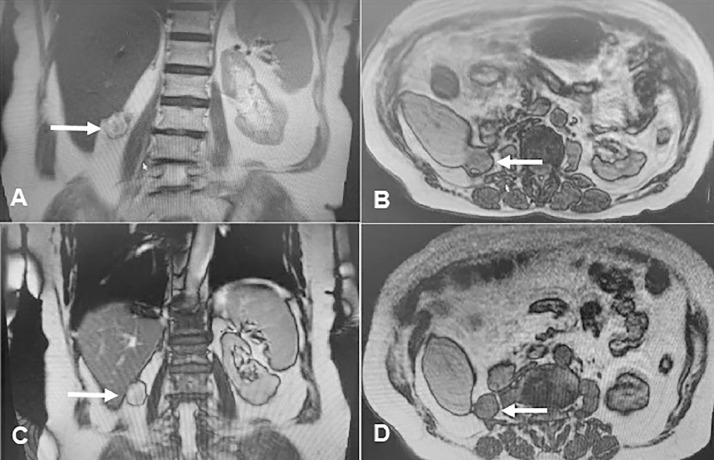
Patient's Abdominopelvic MRI after the surgery. (A and B) 8 month after the surgery. The arrows demonstrate the presence of Surgicel at the surgical site. (C and D) 2 years after the surgery. The arrows indicate the presence of Surgicel at the surgical site, with a smaller size compared to the initial imaging.

Eight months after the surgery, the patient underwent an MRI of the abdomen and pelvis. At the site of the nephrectomy, a lesion measuring 29 × 28 mm was identified, with a loss of the fat plane between it and the liver, but without any signs of diffusion restriction, which is characteristic of a cystic lesion. One year after surgery, during a CT scan of the abdomen and pelvis with intravenous contrast, there was evidence of fat stranding and fluid in the surgical bed, as well as a smooth-walled structure that extended up to the liver capsule and suggested a hematoma. Two years after the surgery, the patient once again underwent an MRI of the abdomen and pelvis, which revealed the presence of the same lesion with smaller dimensions. The MRI report indicated that this finding was consistent with a hematoma (Fig. 1).

## Discussion

The persistance and non-absorption of Surgicel has been discussed in several studies. The absorption of Surgicel is dependent on various factors, such as the amount used, the extent of saturation with blood, and the nature of the surrounding tissue. This process can take several months. Recognizing the radiological manifestations of Surgicel can prevent unnecessary invasive procedures after surgery. Misdiagnoses, such as abscesses, hematomas, or tumor recurrences, can lead to additional invasive procedures, increase patient costs and worry [5].

Knowing the presence of Surgicel at the surgical site is crucial for radiologists and can significantly decrease misdiagnoses. A study conducted in 2023 by Frati and colleagues demonstrated that when radiologists are unaware of the presence of Sergicel, only 11% of potential diagnoses in postoperative CT scans are identified as remnants of the material. However, when radiologists are informed about the presence of Surgicel, 83% of postoperative CT scan reports accurately report its presence, thereby reducing incorrect diagnoses such as hematoma, abscess, or lymphocele [1].

The typical appearance of Surgicel on a postoperative CT scan is characterized by air trapped bubbles. It does not exhibit an air-fluid level or contrast enhancement. If any contrast enhancement is observed, alternative diagnoses should be considered. When Surgicel is used at the surgical site, air becomes trapped, and when blood surrounds the air and gauze, it creates a linear or unifocal punctate pattern [6]. In contrast, an abscess usually manifests as a distinct air-fluid surface with scattered air bubbles. However, if there is any uncertainty, it is advisable to consider measures like broad-spectrum antibiotic therapy and drainage to aid in diagnosing an abscess in a CT scan [6,7].

Having knowledge about the placement of Surgicel at the surgical site, along with the absence of contrast enhancement, proved to be beneficial in making an accurate diagnosis. With regards to the typical presentation of Surgicel in MRI, it exhibits a short relaxation time on T2-weighted images, resulting in low signal intensity in the early postoperative period. On the other hand, an abscess usually demonstrates a signal decrease in T1 and a heterogeneous signal increase in T2. Hence, MRI can be a valuable tool in differentiating between Surgicel and an abscess, consequently avoiding unnecessary interventions. The distribution and appearance of air within Surgicel can appear similar to its appearance on a CT scan [8,9]. In MRI, a hematoma at the surgical site can lead to a signal decrease in T1 and an increase in signal intensity or a heterogeneous signal in T2. Taking into account the clinical findings and changes in size over time can assist in achieving a more accurate diagnosis [9,10]. In our study, the patient underwent MRI during her follow-up, and exhibited the presence of a hematoma. Considering the presence of Surgicel at the surgical site and the elapsed time since surgery, it would be prudent to consider the possibility of Surgicel still being present.

In a study carried out by Oto and his colleagues on patients who underwent cryoablation for kidney tumors and subsequently had Surgicel placed, it was demonstrated that the hypo-intensity of surgicel in T2 images can aid in distinguishing Surgicel from abscesses and hematomas. As a result, the most beneficial paraclinical image for distinguishing Surgicel from an abscess is T2 MRI images. While the margin of Surgicel may not be well defined in T1 phase images, in T2, it has a clear and distinguishable round margin from its surroundings. Unlike tumor recurrence or active bleeding, Surgicel will not absorb contrast material [8]. In another study published by Staglianò and his colleagues in 2018, it was demonstrated that in the evaluation of Surgicel following intracranial surgeries, the findings from diffusion-weighted MRI will provide the most accurate diagnosis [11]. Therefore, performing an MRI and assessing the clinical conditions and time interval since the surgery can be highly beneficial.

## Conclusion

In many cases, radiological examinations will be needed after surgery to check possible complications and also for follow-up. It is important to differentiate the remaining Surgicel from cases such as hematoma, abscess, and tumor recurrence. In these cases, it will be very helpful to inform the radiologist about the presence of Surgicel in the surgical site. Also, T2MRI images will be most accurate in the correct diagnosis of Surgicel.

## Patient consent

Patient informed consent was obtained to publish her information. The patient's private information remained confidential with the researchers.

## CRediT authorship contribution statement

**Anahita Ansari Djafari:** Conceptualization, Methodology, Visualization, Resources, Funding acquisition. **Seyyed Ali Hojjati:** Writing – review & editing, Project administration. **Amir Hossein Eslami:** Supervision, Validation. **Sina Samenezhad:** Data curation, Investigation, Methodology. **Saba Faraji:** Writing – original draft, Software, Formal analysis.
